# Psychoactive prescription drug use and misuse in patients on opioid agonist treatment

**DOI:** 10.1002/bcp.70306

**Published:** 2025-10-16

**Authors:** Thomas Soeiro, Clémence Lacroix, Élisabeth Jouve, Élisabeth Frauger, Maryse Lapeyre‐Mestre, Joëlle Micallef

**Affiliations:** ^1^ UMR 1106, INS, Inserm, Aix‐Marseille University Marseille France; ^2^ Centre for Evaluation and Information on Drug Dependence, Department of Clinical Pharmacology and Drug Surveillance Marseille University Hospital Marseille France; ^3^ Clinical Investigation Center 1436, Team PEPSS, Inserm Toulouse University Hospital, Toulouse University Toulouse France; ^4^ Centre for Evaluation and Information on Drug Dependence, Department of Medical and Clinical Pharmacology Toulouse University Hospital Toulouse France

**Keywords:** addictovigilance, opioid agonist treatment, prescription drug abuse, prescription drug misuse, psychoactive prescription drugs

## Abstract

**Aims:**

To identify the patterns and trends in prescription drug use and misuse in patients on opioid agonist treatment.

**Methods:**

We used data from the OPPIDUM programme, which collects data from patients attending substance abuse treatment facilities. Data collected include use of psychoactive prescription drugs in the past week. In this cross‐sectional study, we included patients aged at least 18 years, on opioid agonist treatment and reporting psychoactive prescription drug use in the past week from 2014 to 2023. The outcome was psychoactive prescription drug misuse (i.e., abuse and/or dependence, illegal acquisition and diverted route of administration) in the past week. We conducted disproportionality analyses to identify prescription drugs associated with misuse. We calculated the prevalence of use and misuse for each prescription drug to estimate trends.

**Results:**

We included 9631 patients. Misuse was disproportionately reported for morphine (e.g., diverted route of administration: *n =* 580; reporting odds ratio: 224.4 [95% confidence interval: 178.8, 281.7]), methylphenidate (e.g., diverted route of administration: 149; 31.6 [24.1, 41.4]), oxycodone (e.g., diverted route of administration: 24; 20.2 [11.1, 36.8]), clonazepam (e.g., illegal acquisition: 48; 6.0 [4.0, 9.0]) and fentanyl (e.g., diverted route of administration: 6; 5.8 [2.3, 14.8]). Trends in misuse paralleled trends in use for most prescription drugs. The sharpest increase in misuse included abuse and/or dependence (+2366%, from 0.9 per 1000 patients in 2014 to 23.0 per 1000 patients in 2023) for pregabalin. Conversely, the sharpest decrease in misuse included illegal acquisition (−59%, from 82.1 per 1000 patients in 2014 to 33.9 per 1000 patients in 2023) for morphine.

**Conclusion:**

In this population, prescription drug misuse primarily included opioid analgesics and increasingly pregabalin. Given the risk of opioid overdose, access to take‐home naloxone should be further improved.

**What is already known about this subject**

- Patients on opioid agonist treatment commonly present with psychiatric and physical comorbidities, often resulting in use of other prescription drugs.
- Many of these drugs also have a high potential for misuse, raising further concerns in this population already vulnerable to substance use disorders.

**What the study adds**

- Prescription drug misuse primarily included opioid analgesics (i.e., morphine, oxycodone and fentanyl), methylphenidate and clonazepam.
- Trends in misuse paralleled trends in use for most prescription drugs.
- Pregabalin misuse increased sharply from 2014 to 2023, while morphine misuse decreased but remained the most common.

## 1 INTRODUCTION

Opioid use disorder is a major public health issue that has developed for decades in many countries worldwide.^1^ Managing patients with opioid use disorder requires an integrated approach that includes medical, social and psychological care. Opioid agonist treatment (OAT, i.e., methadone, buprenorphine and buprenorphine/naloxone combinations) is currently the most effective, evidence‐based pharmacological treatment for opioid use disorder.^2^ OAT decreases cravings and withdrawal symptoms, enabling patients to achieve physical and psychological stability. Long‐term OAT engagement is associated with decreased risk of overdose, all‐cause mortality, infection with hepatitis C virus and human immunodeficiency virus, and improvement in quality of life.^3^, ^4^

Patients on OAT commonly present with psychiatric and physical comorbidities,^5^ often resulting in use of other prescription drugs. Each year, about 50% of patients on OAT use benzodiazepines, Z‐drugs, gabapentinoids, opioid analgesics or stimulants.^6^ Concomitant use of OAT and these drugs is associated with drug–drug interactions and increased mortality, including fatal and nonfatal overdoses.^7^, ^8^ Many of these drugs also have a high potential for misuse, raising further concerns (e.g., increased risk of overdose and development of dependence) in this population already vulnerable to substance use disorders.

In France, OATs have been marketed since the mid‐1990s. Buprenorphine and buprenorphine/naloxone combinations can be initiated by general practitioners, while methadone must be initiated by addiction specialists.^9^ Both can be renewed by general practitioners. This policy of facilitated access has resulted in higher OAT coverage in France than in other European countries, with an estimated 85% coverage rate.^10^ However, methadone is the leading cause of fatal overdoses in France, often in combination with other prescription drugs.^8^ In this context, it is crucial to better understand the current status and dynamics of prescription drug use and misuse in patients on OAT. Therefore, we aimed to identify the patterns and trends in prescription drug use and misuse in patients on OAT in France from 2014 to 2023.

## 2 METHODS

### 2.1 Data source

We used data from the OPPIDUM programme, a multicentre survey conducted every October since 1995 by the French Addictovigilance Network.^11^ The OPPIDUM programme includes patients attending substance abuse treatment facilities (e.g., addiction care centres, harm reduction centres, outpatient addiction services, inpatient addiction services, liaison teams for addiction care and addiction care units in prison) distributed throughout France. Since 2010, about 5000 patients have been included each year across about 700 substance abuse treatment facilities. Participation of substance abuse treatment facilities and patients to the OPPIDUM programme is voluntary. Data from the OPPIDUM programme have previously provided valuable insights into OAT use in France.^12^, ^13^

Patients are interviewed using anonymous, standardized questionnaires. Data collected include: patients' sociodemographic characteristics (e.g., age, sex, partner, children, education, employment and housing); OAT use; naloxone availability; substance abuse treatment facility attended; use of alcohol and tobacco; and use of illicit drugs and psychoactive prescription drugs in the past week (e.g., frequency, dose, onset, abuse and/or dependence, source of supply and route of administration). Prescription drugs are coded using the Anatomical Therapeutic Chemical classification system.

### 2.2 Study population

In this cross‐sectional study, we included patients aged at least 18 years, on OAT (i.e., methadone, buprenorphine or buprenorphine/naloxone combinations) and reporting psychoactive prescription drug use in the past week from 2014 to 2023. Psychoactive prescription drugs were defined as drugs acting on the central nervous system, including anaesthetics (N01), analgesics (N02), antiepileptics (N03), anti‐Parkinson drugs (N04), psycholeptics (N05), psychoanaleptics (N06) and other nervous system drugs (N07).

### 2.3 Outcome

The outcome was psychoactive prescription drug misuse in the past week, assessed using 3 misuse behaviours: abuse and/or dependence according to the Diagnostic and Statistical Manual of Mental Disorders^14^; illegal acquisition (e.g., by doctor shopping,^15^ forged prescription, dealing, gifting, theft or online purchases); and diverted route of administration (i.e., route of administration not authorised in the summary of product characteristics).

### 2.4 Analyses

For the analyses, we grouped buprenorphine and buprenorphine/naloxone combinations due to the limited use of buprenorphine/naloxone combinations in France.

First, we described the study population, both overall and for each year from 2014 to 2023.

Second, we calculated the prevalence of use for each prescription drug separately, both overall and for each year from 2014 to 2023. We used the number of patients included each year as the denominator. We calculated the percentage change from 2014 to 2023 to estimate trends.

Third, we conducted disproportionality analyses to identify prescription drugs associated with misuse. We calculated the reporting odds ratio (ROR) for each prescription drug separately as the odds of the drug being mentioned in reports with a given misuse behaviour divided by the odds of the drug being mentioned in reports without the misuse behaviour.^16^ An ROR was considered statistically significant if the lower bound of the 95% confidence interval was >1, indicating that the misuse behaviour was disproportionately reported for the drug. We conducted subgroup analyses in patients on methadone and in patients on buprenorphine ± naloxone. Disproportionality analyses have previously been applied to assess prescription drug misuse.^17^, ^18^

Finally, we calculated the prevalence of misuse behaviours for each prescription drug separately for each year from 2014 to 2023, focusing on the pairs of misuse behaviours and prescription drugs that were disproportionately reported. Again, we used the number of patients included each year as the denominator. We fitted local polynomial regression curves and calculated the percentage change from 2014 to 2023 to estimate trends.

We conducted analyses using R version 4.4.0 and generated figures using ggplot2 version 3.5.1.^19^, ^20^

### 2.5 Ethics

The OPPIDUM programme and subsequent analyses do not require regulatory authorisation because the data collected are anonymous. The reporting of the study adheres to the Strengthening the Reporting of Observational Studies in Epidemiology statement.^21^

### 2.6 Nomenclature of targets and ligands

Key protein targets and ligands in this article are hyperlinked to corresponding entries in http://www.guidetopharmacology.org and are permanently archived in the Concise Guide to PHARMACOLOGY 2021/22.^22^

## 3 RESULTS

### 3.1 Study population

**FIGURE 1:**
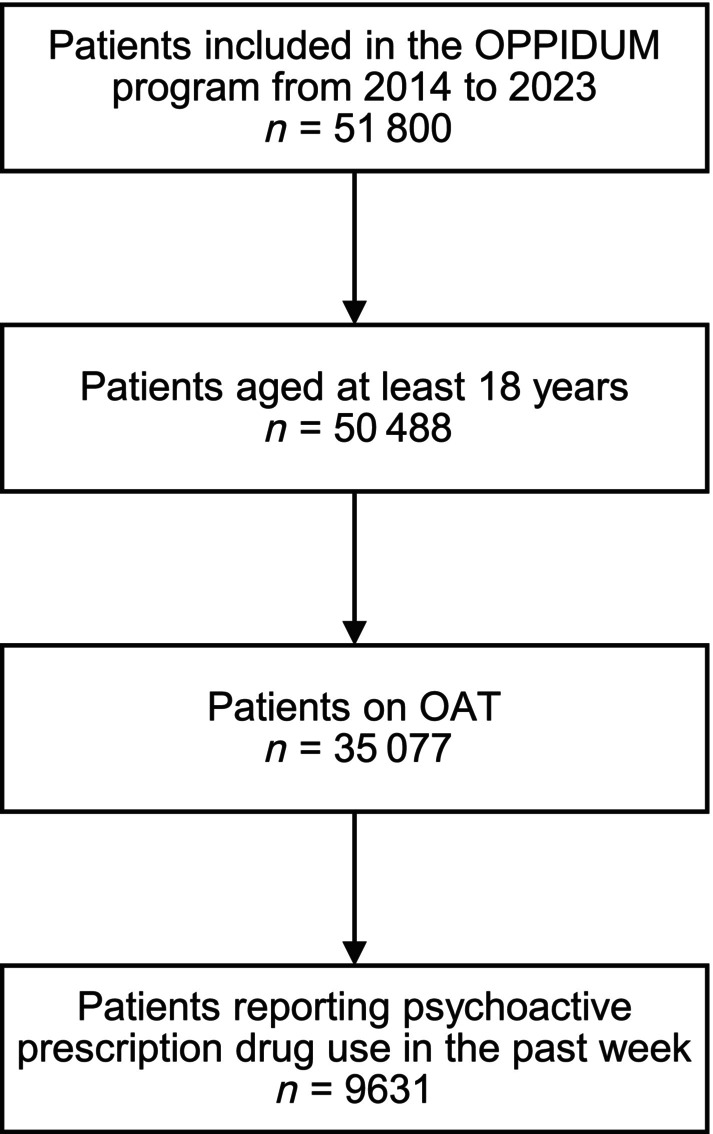
Study flowchart.

Among the 51 800 patients included in the OPPIDUM programme from 2014 to 2023, we included 9631 patients (Figure 1). Apart from OAT, the patients provided 15 884 reports of prescription drug use for 111 different prescription drugs. The patients attended 510 substance abuse treatment facilities.

The median age was 41 [34, 48] year and 78% of patients were men (Table 1). Regarding social status, 25% had a partner and 21% had children. Regarding economic status, 61% had a technical education, 23% were employed and 77% had stable housing. Regarding OAT and naloxone, 69% were on methadone, 31% were on buprenorphine ± naloxone and 25% had naloxone available. Regarding substance use, 31% had alcohol dependence, 92% used tobacco, 10% used heroine, 29% used cannabis, 20% used cocaine and 3% used designer drugs.

**TABLE 1:** Study population.

	2014	2015	2016	2017	2018	2019	2020	2021	2022	2023	Total
Number of patients	1072	1063	1067	1009	1038	995	826	884	851	826	9631
Number reports of prescription drug use	1845	1866	1774	1634	1678	1655	1345	1431	1389	1267	15 884
Age in years, median [IQR]	38 [32, 45]	40 [33, 45]	40 [33, 46]	41 [34, 48]	40 [34, 48]	42 [35, 48]	41 [35, 48]	43 [36, 50]	44 [37, 50]	44 [38, 51]	41 [34, 48]
Men, *n* (%)	819 (77)	835 (79)	850 (80)	790 (79)	801 (78)	774 (78)	624 (76)	705 (80)	650 (77)	631 (77)	7479 (78)
Having a partner, *n* (%)	300 (28)	270 (26)	248 (24)	260 (26)	263 (25)	239 (24)	199 (24)	206 (24)	194 (23)	176 (22)	2355 (25)
Having children, *n* (%)	240 (23)	208 (20)	198 (19)	207 (21)	222 (22)	201 (21)	176 (22)	175 (20)	175 (21)	172 (21)	1974 (21)
Education, *n* (%)											
Primary school	60 (6)	63 (6)	79 (8)	57 (6)	56 (6)	45 (5)	48 (6)	28 (3)	31 (4)	33 (4)	500 (5)
Technical education	639 (62)	629 (62)	650 (63)	602 (61)	618 (62)	578 (61)	452 (57)	527 (63)	495 (60)	488 (62)	5678 (61)
High school	224 (22)	214 (21)	198 (19)	215 (22)	228 (23)	208 (22)	178 (22)	178 (21)	187 (23)	156 (20)	1986 (21)
Higher education	101 (10)	107 (11)	111 (11)	105 (11)	99 (10)	118 (12)	118 (15)	101 (12)	111 (13)	109 (14)	1080 (12)
Employed, *n* (%)	246 (24)	210 (20)	222 (21)	233 (24)	231 (23)	217 (22)	190 (23)	211 (24)	206 (25)	185 (23)	2151 (23)
Stable housing, *n* (%)	784 (78)	775 (77)	786 (78)	759 (79)	758 (77)	709 (73)	617 (77)	672 (78)	651 (78)	612 (76)	7123 (77)
Opioid agonist treatment, *n* (%)											
Buprenorphine ± naloxone	285 (27)	336 (32)	324 (30)	335 (33)	359 (35)	329 (33)	224 (27)	249 (28)	280 (33)	275 (33)	2996 (31)
Methadone	787 (73)	727 (68)	743 (70)	674 (67)	679 (65)	666 (67)	602 (73)	635 (72)	571 (67)	551 (67)	6635 (69)
Naloxone available, *n* (%)	0 (0)	0 (0)	0 (0)	0 (0)	0 (0)	164 (17)	186 (23)	240 (28)	234 (29)	253 (31)	1077 (25)
Substance abuse treatment facility, *n* (%)											
Addiction care centre	817 (76)	927 (87)	841 (79)	762 (76)	754 (73)	711 (71)	595 (72)	633 (72)	642 (75)	597 (72)	7279 (76)
Harm reduction centre	101 (9)	44 (4)	102 (10)	92 (9)	108 (10)	124 (12)	81 (10)	99 (11)	87 (10)	88 (11)	926 (10)
Outpatient addiction service	71 (7)	52 (5)	55 (5)	47 (5)	47 (5)	43 (4)	78 (9)	85 (10)	61 (7)	73 (9)	612 (6)
Inpatient addiction service	4 (0)	5 (0)	10 (1)	14 (1)	9 (1)	27 (3)	16 (2)	3 (0)	9 (1)	22 (3)	119 (1)
Liaison team for addiction care	7 (1)	5 (0)	27 (3)	48 (5)	46 (4)	23 (2)	21 (3)	24 (3)	28 (3)	10 (1)	239 (2)
Addiction care unit in prison	53 (5)	26 (2)	18 (2)	42 (4)	67 (6)	58 (6)	26 (3)	30 (3)	24 (3)	29 (4)	373 (4)
Other centre	19 (2)	4 (0)	14 (1)	4 (0)	7 (1)	9 (1)	9 (1)	10 (1)	0 (0)	7 (1)	83 (1)
Alcohol dependence, *n* (%)	297 (28)	289 (28)	316 (30)	303 (30)	323 (31)	312 (32)	258 (32)	297 (34)	280 (33)	274 (34)	2949 (31)
Tobacco use, *n* (%)	995 (94)	987 (94)	989 (94)	912 (92)	951 (92)	893 (91)	749 (91)	796 (91)	752 (92)	735 (90)	8759 (92)
Heroin use, *n* (%)	110 (10)	53 (5)	96 (9)	105 (10)	94 (9)	117 (12)	96 (12)	98 (11)	90 (11)	81 (10)	940 (10)
Cannabis use, *n* (%)	295 (28)	330 (31)	322 (30)	315 (31)	307 (30)	277 (28)	214 (26)	258 (29)	225 (26)	230 (28)	2773 (29)
Cocaine use, *n* (%)	134 (12)	116 (11)	185 (17)	196 (19)	226 (22)	248 (25)	200 (24)	185 (21)	204 (24)	234 (28)	1928 (20)
Designer drug use, *n* (%)	34 (3)	24 (2)	42 (4)	36 (4)	32 (3)	28 (3)	31 (4)	21 (2)	9 (1)	23 (3)	280 (3)

Most patients' characteristics were relatively stable from 2014 to 2023, except a sharp increase in cocaine use (from 12% in 2014 to 28% in 2023) and having naloxone available (from 17% in 2019 to 31% in 2023, after naloxone was marketed in France). To a lesser extent, age (from 38 [32, 45] in 2014 to 44 [38, 51] in 2023), higher education (from 10% in 2014 to 14% in 2023) and alcohol dependence (from 28% in 2014 to 34% in 2023) also increased. Conversely, having a partner (from 28% in 2014 to 22% in 2023) and tobacco use (from 94% in 2014 to 90% in 2023) decreased.

### 3.2 Trends in the prevalence of prescription drug use

Apart from OAT, the prescription drugs with the highest prevalence of use included (Table 2): anxiolytics, primarily diazepam (237.8 per 1000 patients) and oxazepam (213.6 per 1000 patients); hypnotics and sedatives, primarily zopiclone (97.0 per 1000 patients) and zolpidem (50.7 per 1000 patients); antipsychotics, primarily cyamemazine (79.1 per 1000 patients) and olanzapine (43.3 per 1000 patients); opioid analgesics, primarily morphine (72.3 per 1000 patients) and tramadol (10.7 per 1000 patients); and antidepressants, primarily venlafaxine (46.5 per 1000 patients) and paroxetine (40.8 per 1000 patients). Other prescription drugs with high prevalence of use were methylphenidate (25.0 per 1000 patients) and pregabalin (15.9 per 1000 patients).

**TABLE 2:** Trends in the prevalence of prescription drug use per 1000 patients. Data not shown for prescription drugs with a prevalence of use of <10 per 1000 patients.

	2014	2015	2016	2017	2018	2019	2020	2021	2022	2023	Total	Percentage change from 2014 to 2023
Diazepam (N05BA01)	190.3	240.8	261.5	235.9	245.7	261.3	232.4	225.1	251.5	233.7	237.8	+23%
Oxazepam (N05BA04)	214.6	210.7	183.7	199.2	212.9	214.1	211.9	230.8	232.7	236.1	213.6	+10%
Zopiclone (N05CF01)	111.0	94.1	101.2	111.0	95.4	96.5	78.7	87.1	96.4	92.0	97.0	−17%
Cyamemazine (N05AA06)	97.9	100.7	89.0	80.3	81.9	76.4	56.9	74.7	57.6	61.7	79.1	−37%
Morphine (N02AA01)	98.9	79.0	86.2	81.3	68.4	69.3	64.2	65.6	51.7	44.8	72.3	−55%
Alprazolam (N05BA12)	68.1	56.4	54.4	65.4	66.5	74.4	83.5	82.6	77.6	76.3	69.7	+12%
Bromazepam (N05BA08)	84.0	86.5	75.0	67.4	64.5	44.2	73.8	69.0	61.1	66.6	69.6	−21%
Zolpidem (N05CF02)	79.3	59.3	73.1	52.5	49.1	37.2	32.7	46.4	36.4	26.6	50.7	−66%
Venlafaxine (N06AX16)	48.5	52.7	33.7	42.6	47.2	43.2	48.4	56.6	49.4	44.8	46.5	−8%
Olanzapine (N05AH03)	42.9	50.8	45.9	53.5	45.3	38.2	41.2	47.5	38.8	24.2	43.3	−44%
Paroxetine (N06AB05)	33.6	42.3	42.2	42.6	32.8	44.2	49.6	45.2	40.0	37.5	40.8	+12%
Aripiprazole (N05AX12)	42.0	40.5	38.4	35.7	46.2	46.2	48.4	31.7	40.0	33.9	40.4	−19%
Quetiapine (N05AH04)	26.1	24.5	30.9	31.7	40.5	47.2	41.2	43.0	45.8	43.6	36.9	+67%
Mirtazapine (N06AX11)	20.5	26.3	43.1	36.7	30.8	32.2	33.9	36.2	37.6	48.4	34.2	+136%
Lormetazepam (N05CD06)	29.9	32.9	36.6	39.6	30.8	26.1	32.7	43.0	28.2	21.8	32.3	−27%
Mianserin (N06AX03)	20.5	34.8	28.1	25.8	37.6	39.2	27.8	31.7	42.3	29.1	31.6	+42%
Hydroxyzine (N05BB01)	68.1	62.1	39.4	30.7	12.5	14.1	15.7	17.0	11.8	20.6	30.5	−70%
Prazepam (N05BA11)	22.4	21.6	29.1	28.7	28.9	43.2	25.4	19.2	48.2	36.3	30.0	+62%
Loxapine (N05AH01)	21.5	36.7	30.0	28.7	37.6	32.2	32.7	21.5	27.0	25.4	29.5	+18%
Risperidone (N05AX08)	28.9	42.3	24.4	23.8	22.2	24.1	25.4	18.1	28.2	19.4	26.0	−33%
Methylphenidate (N06BA04)	25.2	21.6	16.9	22.8	30.8	25.1	32.7	28.3	22.3	26.6	25.0	+6%
Sertraline (N06AB06)	19.6	24.5	16.9	12.9	18.3	22.1	23.0	29.4	50.5	23.0	23.5	+17%
Fluoxetine (N06AB03)	34.5	20.7	17.8	14.9	13.5	22.1	21.8	26.0	27.0	33.9	22.9	−2%
Escitalopram (N06AB10)	58.8	29.2	20.6	22.8	15.4	10.1	12.1	11.3	5.9	10.9	20.7	−81%
Pregabalin (N02BF02)	8.4	6.6	5.6	8.9	6.7	22.1	24.2	20.4	28.2	37.5	15.9	+347%
Amitriptyline (N06AA09)	9.3	14.1	17.8	11.9	27.9	18.1	19.4	15.8	11.8	12.1	15.9	+30%
Potassium clorazepate (N05BA05)	31.7	16.0	12.2	11.9	9.6	19.1	18.2	11.3	16.5	8.5	15.7	−73%
Lorazepam (N05BA06)	12.1	16.9	10.3	10.9	11.6	12.1	10.9	9.0	15.3	13.3	12.3	+10%
Tramadol (N02AX02)	3.7	7.5	6.6	6.9	10.6	21.1	14.5	14.7	11.8	12.1	10.7	+224%
Duloxetine (N06AX21)	16.8	16.9	12.2	10.9	10.6	11.1	7.3	4.5	4.7	4.8	10.4	−71%

The sharpest increase in use included pregabalin (+347%, from 8.4 per 1000 patients in 2014 to 37.5 per 1000 patients in 2023), tramadol (+224%, from 3.7 per 1000 patients in 2014 to 12.1 per 1000 patients in 2023), oxycodone (+224%, from 1.9 per 1000 patients in 2014 to 6.1 per 1000 patients in 2023) and fentanyl (160%, from 0.9 per 1000 patients in 2014 to 2.4 per 1000 patients in 2023). Conversely, the sharpest decrease in use included escitalopram (−81%, from 58.8 per 1000 patients in 2014 to 10.9 per 1000 patients in 2023), hydroxyzine (−70%, from 68.1 per 1000 patients in 2014 to 20.6 per 1000 patients in 2023), zolpidem (−66%, from 79.3 per 1000 patients in 2014 to 26.6 per 1000 patients in 2023) and morphine (−55%, from 98.9 per 1000 patients in 2014 to 44.8 per 1000 patients in 2023).

### 3.3 Prescription drugs associated with misuse

**FIGURE 2:**
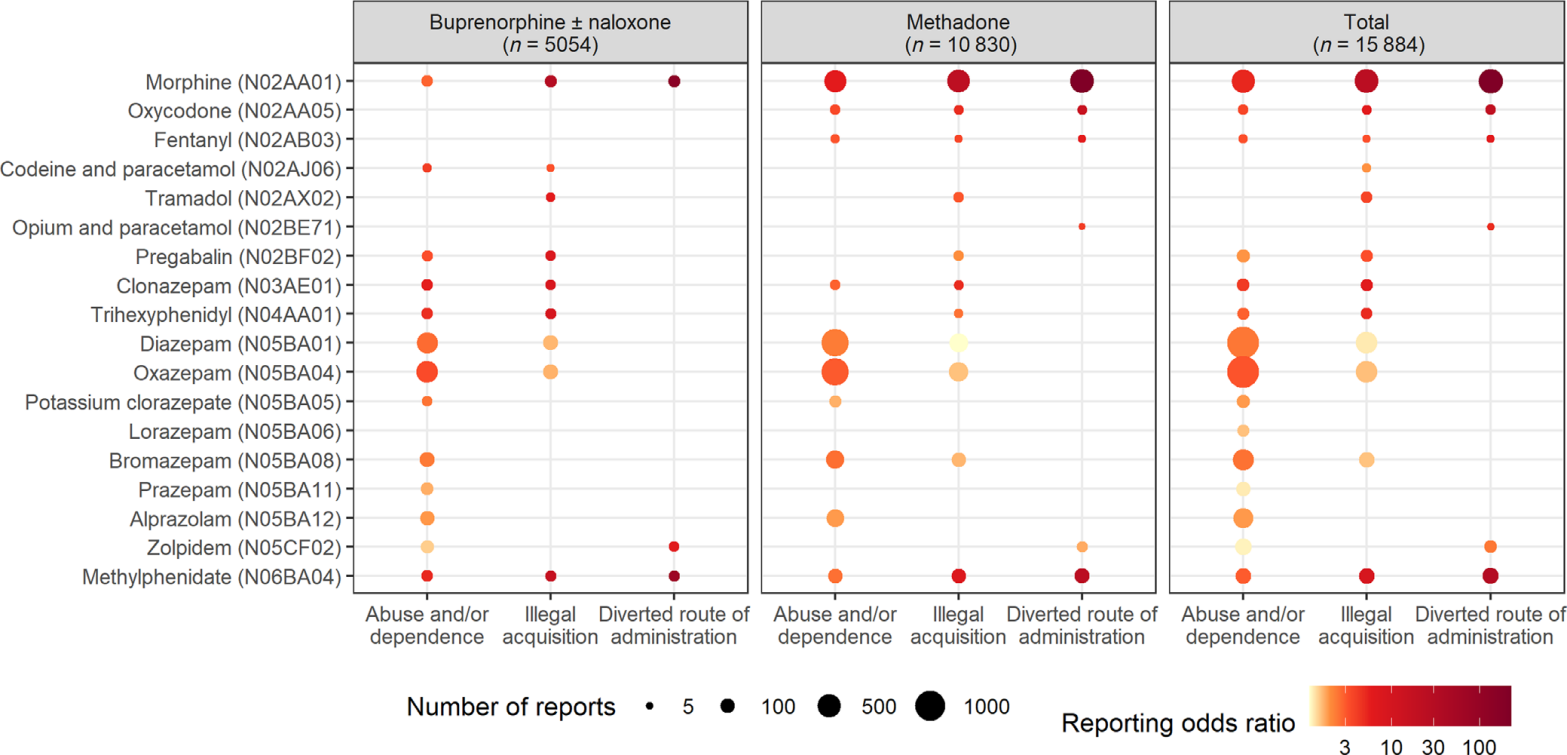
Prescription drugs associated with misuse.

Among the 15 884 reports of prescription drug use, 5235 (33%) mentioned abuse and/or dependence, 2292 (14%) mentioned illegal acquisition and 911 (6%) mentioned diverted route of administration. Misuse was disproportionately reported for 18 prescription drugs (Figure 2). Some of the highest RORs included: diverted route of administration (*n =* 580; ROR: 224.4 [95% confidence interval: 178.8, 281.7]), illegal acquisition (530; 24.3 [20.3, 29.2]) and abuse and/or dependence (487; 5.1 [4.3, 6.0]) for morphine; diverted route of administration (149; 31.6 [24.1, 41.4]), illegal acquisition (142; 9.0 [6.9, 11.7]) and abuse and/or dependence (141; 2.9 [2.3, 3.8]) for methylphenidate; diverted route of administration (24; 20.2 [11.1, 36.8]), illegal acquisition (22; 6.0 [3.3, 10.8]) and abuse and/or dependence (28; 3.6 [1.9, 6.6]) for oxycodone; illegal acquisition (48; 6.0 [4.0, 9.0]) and abuse and/or dependence (64; 4.1 [2.7, 6.3]) for clonazepam; and diverted route of administration (6; 5.8 [2.3, 14.8]), illegal acquisition (8; 3.2 [1.3, 7.5]) and abuse and/or dependence (14; 3.2 [1.4, 7.3]) for fentanyl. Additionally, diverted route of administration was disproportionately reported for opium/paracetamol combinations (5; 4.9 [1.8, 13.2]) and zolpidem (59; 2.3 [1.8, 3.1]); illegal acquisition for trihexyphenidyl (43; 5.0 [3.3, 7.5]), tramadol (39; 3.7 [2.5, 5.5]) and pregabalin (53; 3.2 [2.3, 4.5]); and abuse and/or dependence for oxazepam (1158; 3.1 [2.8, 3.4]), bromazepam (360; 2.5 [2.1, 2.9]) and diazepam (1155; 2.4 [2.2, 2.6]).

In subgroup analyses, morphine had higher RORs for diverted route of administration and abuse and/or dependence in patients on methadone than in patients on buprenorphine ± naloxone (Figure 2). Additionally, misuse was disproportionately reported for oxycodone and fentanyl in patients on methadone but not in patients on buprenorphine ± naloxone. Conversely, most other prescription drugs had higher RORs in patients on buprenorphine ± naloxone than in patients on methadone.

### 3.4 Trends in the prevalence of prescription drug misuse

**FIGURE 3:**
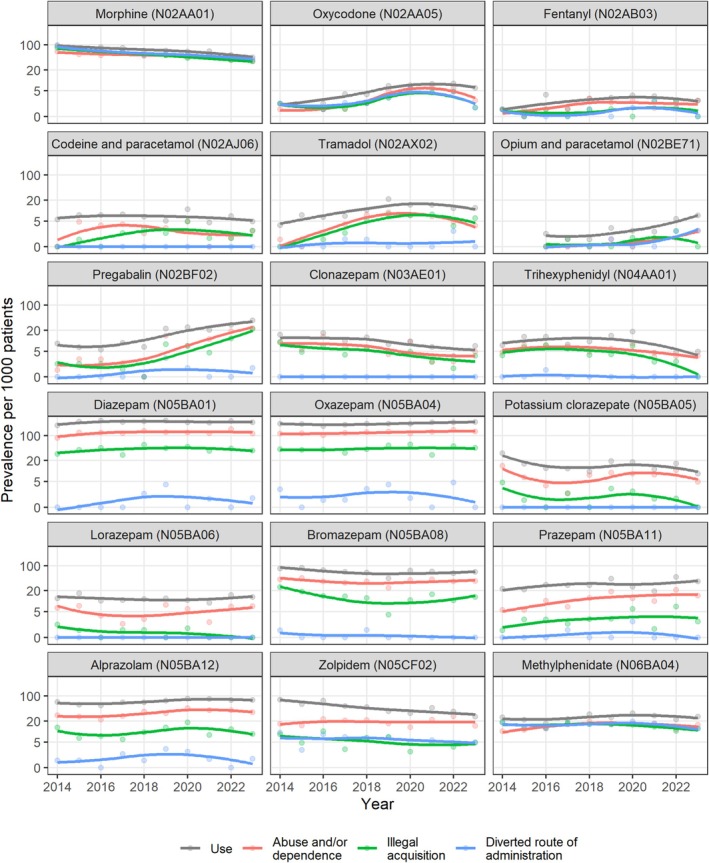
Trends in the prevalence of prescription drug use and misuse.

Trends in misuse paralleled trends in use for most prescription drugs (Figure 3). The sharpest increase in misuse included: abuse and/or dependence (+2366%, from 0.9 per 1000 patients in 2014 to 23.0 per 1000 patients in 2023) and illegal acquisition (+1068%, from 1.9 per 1000 patients in 2014 to 21.8 per 1000 patients in 2023) for pregabalin; illegal acquisition (from no patients in 2014 to 6.1 per 1000 patients in 2023) and abuse and/or dependence (+289%, from 0.9 per 1000 patients in 2014 to 3.6 per 1000 patients in 2023) for tramadol; abuse and/or dependence (+160%, from 5.6 per 1000 patients in 2014 to 14.5 per 1000 patients in 2023) and illegal acquisition (+160%, from 0.9 per 1000 patients in 2014 to 2.4 per 1000 patients in 2023) for prazepam; illegal acquisition (from no patients in 2014 to 2.4 per 1000 patients in 2023) for codeine/paracetamol combinations; and abuse and/or dependence for oxycodone and fentanyl (+160%, from 0.9 per 1000 patients in 2014 to 2.4 per 1000 patients in 2023 for both). After peaking in 2020–2022, oxycodone misuse decreased in 2023.

Conversely, the sharpest decrease in misuse included: illegal acquisition (−59%, from 82.1 per 1000 patients in 2014 to 33.9 per 1000 patients in 2023), diverted route of administration (−58%, from 91.4 per 1000 patients in 2014 to 38.7 per 1000 patients in 2023) and abuse and/or dependence (−45%, from 64.4 per 1000 patients in 2014 to 35.1 per 1000 patients in 2023) for morphine; illegal acquisition (−61%, from 9.3 per 1000 patients in 2014 to 3.6 per 1000 patients in 2023) and abuse and/or dependence (−48%, from 9.3 per 1000 patients in 2014 to 4.8 per 1000 patients in 2023) for clonazepam; diverted route of administration (−48%, from 9.3 per 1000 patients in 2014 to 4.8 per 1000 patients in 2023) and illegal acquisition (−42%, from 8.4 per 1000 patients in 2014 to 4.8 per 1000 patients in 2023) for zolpidem; illegal acquisition (from 3.7 per 1000 patients in 2014 to no patients in 2023) for trihexyphenidyl; and illegal acquisition (−35%, from 18.7 per 1000 patients in 2014 to 12.1 per 1000 patients in 2023) and diverted route of administration (−32%, from 17.7 per 1000 patients in 2014 to 12.1 per 1000 patients in 2023) for methylphenidate.

## 4 DISCUSSION

The study describes the patterns and trends in prescription drug use and misuse in patients on OAT. While the prevalence of use of anxiolytics, antipsychotics and antidepressants was high, prescription drug misuse primarily included opioid analgesics (i.e., morphine, oxycodone and fentanyl), methylphenidate and clonazepam. Trends in misuse paralleled trends in use for most prescription drugs. In particular, pregabalin misuse increased sharply from 2014 to 2023, while morphine misuse decreased but remained the most common.

Additionally, 10% of patients used heroin and misuse of opioid analgesics (e.g., morphine, oxycodone and fentanyl) was common in patients on methadone. A previous study also found that OAT, in particular methadone, is associated with ongoing heroin use in France.^23^ Concomitant use of methadone and other opioids increases the risk of sedation, respiratory depression and overdose. However, only 25% of patients had naloxone available. While naloxone may have been prioritized for patients at higher risk of opioid overdose, access to take‐home naloxone should be further improved.

Similarly, use and misuse of gabapentinoids and benzodiazepines in patients on OAT require close monitoring due to the increased risk of opioid overdose.^24^, ^25^ This concern is supported by evidence that pregabalin administration in oxycodone‐dependent mice reverses opioid tolerance and increases the risk of respiratory depression.^26^ A previous study using data from the OPPIDUM programme found that the number of patients on pregabalin in substance use treatment facilities increased 12‐fold from 2008 to 2022, with changes in patients' characteristics suggesting more severe substance use disorders.^27^

Our findings contribute to the monitoring of prescription drug misuse. Addressing prescription drug misuse requires a multimodal monitoring system to identify prescription drug misuse across different populations timely, sensitively and specifically.^28^ The French Addictovigilance Network established and manages such a multimodal monitoring system in France.^29^ Our findings further strengthen evidence of prescription drug misuse in the general population accumulated by the French Addictovigilance Network, in particular for pregabalin,^30^, ^31^ tramadol,^32^ oxycodone^33^ and methylphenidate.^34^, ^35^ While these signals have been sufficiently confirmed, coordinated actions remain necessary to improve the safety of these drugs. Our findings also enable comparative quantification, which may support more targeted regulatory interventions.

Finally, our findings enable assessment of the impact of previous regulatory interventions. In particular, escitalopram and hydroxyzine were contraindicated with methadone in 2016 due to the risk of ventricular rhythm disorder. We found that use of escitalopram and hydroxyzine decreased sharply in patients on OAT in substance abuse treatment facilities, 69% of whom were on methadone, suggesting substantial improvement. Additionally, zolpidem must be prescribed using tamper‐resistant prescription forms since April 2017, resulting in decreased use in the general population.^36^ We found that zolpidem use decreased in patients on OAT in substance abuse treatment facilities, while zolpidem misuse decreased more slowly.

### 4.1 Limitations

The main limitation of the study is the potential for selection bias because participation of substance abuse treatment facilities and patients to the OPPIDUM programme is voluntary. The resulting sample may not be representative of all patients on OAT attending substance abuse treatment facilities. Additionally, some patients may have been included more than once across different years or substance abuse treatment facilities because the data are anonymous. However, to our knowledge, the OPPIDUM programme is unparalleled in describing substance use in this vulnerable population.

## 5 CONCLUSIONS

The study provides an overview of the current status and dynamics of prescription drug use and misuse in this population. Prescription drug misuse primarily included opioid analgesics and increasingly pregabalin. Given the risk of opioid overdose, access to take‐home naloxone should be further improved.

## AUTHOR CONTRIBUTIONS

**Thomas Soeiro:** Conceptualization; formal analysis; methodology; software; visualization; writing—original draft. **Clémence Lacroix:** Data curation; writing—review and editing. **Élisabeth Jouve:** Data curation. **Élisabeth Frauger:** Data curation. **Maryse Lapeyre‐Mestre:** Conceptualization; writing—review and editing. **Joëlle Micallef:** Conceptualization; supervision; writing—review and editing.

## CONFLICT OF INTEREST STATEMENT

We declare no competing interests.

## Data Availability

The data that support the findings of the study are available from the corresponding author upon reasonable request.
